# Body visual discontinuity affects feeling of ownership and skin conductance responses

**DOI:** 10.1038/srep17139

**Published:** 2015-11-25

**Authors:** Gaetano Tieri, Emmanuele Tidoni, Enea Francesco Pavone, Salvatore Maria Aglioti

**Affiliations:** 1 Fondazione Santa Lucia, IRCCS, Rome, Italy; 2 Department of Psychology, University of Rome “La Sapienza”, Rome, Italy; 3 Braintrends ltd, Applied Neuroscience, Rome, Italy

## Abstract

When we look at our hands we are immediately aware that they belong to us and we rarely doubt about the integrity, continuity and sense of ownership of our bodies. Here we explored whether the mere manipulation of the visual appearance of a virtual limb could influence the subjective feeling of ownership and the physiological responses (Skin Conductance Responses, SCRs) associated to a threatening stimulus approaching the virtual hand. Participants observed in first person perspective a virtual body having the right hand-forearm (i) connected by a normal wrist (Full-Limb) or a thin rigid wire connection (Wire) or (ii) disconnected because of a missing wrist (m-Wrist) or a missing wrist plus a plexiglass panel positioned between the hand and the forearm (Plexiglass). While the analysis of subjective ratings revealed that only the observation of natural full connected virtual limb elicited high levels of ownership, high amplitudes of SCRs were found also during observation of the non-natural, rigid wire connection condition. This result suggests that the conscious embodiment of an artificial limb requires a natural looking visual body appearance while implicit reactivity to threat may require physical body continuity, even non-naturally looking, that allows the implementation of protective reactions to threat.

Protective physiological reactions to events threatening our body depend not only on the recognition of the threat itself but also on the correct representation of our body and its attribution to ourselves. Integrating multiple sensory information and solving possible incongruences is fundamental for correctly attributing an external object to our Self. Indeed the synchronous (but not asynchronous) stroking of an external rubber hand with the participant’s hidden hand can induce an illusory Feeling of Ownership (FO) over the external body-part (Rubber Hand Illusion, RHI)^1^ and an increase in Skin Conductance Responses (SCRs) when a threatening stimulus is directed towards the embodied rubber hand^2^.

Previous research highlighted that the illusory FO is affected by the external object’s shape^3^,^4^, anatomical and postural appearance^5^,^6^,^7^,^8^ and by the congruency between multisensory visuo-tactile^1^, visuo-motor^9^,^10^,^11^ and visuo-spatial^12^ information (see^13^ for a recent and comprehensive review of the factors affecting and promoting the illusory body ownership in healthy subjects). Moreover, recent computational models^13^,^14^ and behavioural studies suggest that visual information play a crucial role in modulating the FO over a fake hand in brain damaged^15^,^16^, spinal cord injured people^17^ and in healthy participants when synchronous visuo-tactile stimulation^1^ is applied and when the external object is passively observed^5^,^18^,^19^.

By means of Immersive Virtual Reality (IVR) the original version of the RHI has been readapted to investigate the FO over virtual body parts^20^,^21^,^22^ and full bodies^23^,^24^,^25^ presenting a virtual surrogate at the same location as the participants’ real body (thus with reduced visual, proprioceptive and spatial mismatches between the real and the virtual body). These studies highlighted the crucial role of the first-Person Perspective (1PP)^23^,^24^,^25^, i.e. the observation of the world from the eyes of the virtual body that visually substitutes the person’s real one, and visual continuity of body parts^18^,^26^ to elicit high levels of body ownership even when the virtual body is passively observed^18^,^27^.

Indeed, the unitary visual perception of our body represents a stable information, i.e. we do not experience our body as an unorganized assembly of disowned limbs. Yet, information about the role of body visual (dis)continuity in permeating body representation at conscious and unconscious levels is still limited. Perez-Marcos and colleagues^26^ showed that, during synchronous visuo-tactile stimulation of the virtual and participant’s real hand, the level of ownership over the virtual hand was reduced if physical discontinuity between the virtual hand and shoulder disrupted limb integrity^26^. We recently expanded this study using an IVR set-up in which participants passively observed in 1PP a virtual body that could appear with the right hand progressively detached to the body. We found that, in the absence of multisensory stimulation^26^ and without any alteration of the peripersonal space^28^,^29^, a small visual discontinuity between the virtual hand and body can disrupt the subjective FO over the virtual hand itself^18^.

In the present study we explored whether the subjective experience of owning the virtual hand and the physiological responses (SCRs) associated to a threatening stimulus approaching the virtual hand were modified by the different visual appearances of the virtual limb. In particular, we used an Head Mounted Display (HMD) to immerse healthy participants in a virtual reality scenario where they observed in 1PP a virtual upper limb (Fig. 1A) that could have the four different visual appearances shown in Fig. 1B. Thus in different blocks participants observed: (i) a standard full limb (i.e. with hand connected the rest of the body; Full-Limb); (ii) a limb with a thin black rigid wire connecting the forearm and the hand (Wire); (iii) a limb with a missing wrist (m-Wrist) and (iv) a limb with a Plexiglass panel placed in a blank space between the hand and the forearm (Plexiglass). Importantly the Wire condition maintained a physical connection between the forearm and the hand by substituting the virtual wrist with a thin black rigid wire while the Plexiglass condition emphasised the disconnection between the two body parts.

Based on earlier findings that mere passive observation of a virtual body in 1PP^25^,^30^,^31^ and the integrity of the body’s visual continuity^18^,^26^ are both sufficient conditions in eliciting the illusory FO, we expected the highest subjective ratings of FO during the observation of a full virtual limb and highest SCRs when the full virtual limb was threatened. Moreover, we expected no significant differences in subjective ratings and SCR when participants perceived the non-natural virtual limbs (m-Wrist, Plexiglass and Wire).

## Results

Subjects who exhibited SCRs’ amplitudes lower than .01 μS in more than half of the experimental conditions were considered as ‘null-responders’ and excluded from all subsequent analyses. Based on this criteria one participant was excluded and we evaluated SCRs and questionnaire’s answers of 19 participants.

### Questionnaire

The mean of Q1 and Q2 and the mean of Q3 and Q4 were considered as indexes of the perceived FO over the virtual hand (Hand-FO) and the virtual forearm (Forearm-FO) respectively. Data were analyzed using an ordered logistic regression with Body Part (Hand, Arm) and Visual Appearance (Full-Limb, Wire, m-Wrist, Plexiglass) as within subjects factors.

To select the best GLM (in terms of goodness of fit and factor significance), a series of models, accounting for all factors and their interactions, were computed by considering both Akaike information criterion (AIC), Bayesian information criterion (BIC) and the log likelihood indexes.

The model fitted with Visual Appearance as single predictor explained better the variability of our data (see Table 1). Importantly, the non-significant effect of the remaining models (third and fourth row Table 1) suggests that body visual discontinuity affects the FO over the whole limb, independently from the specific body part. Moreover participants experienced higher levels of body ownership during the Full-Limb (median value ± interquartile range, 5.25 ± 1.50) relative to other experimental conditions (m-Wrist, 3.50 ± 1.50; Plexiglass, 4.50 ± 1.875; Wire, 4.00 ± 1.875) (see Table 2 top row). The remaining contrasts revealed no differences between the other visual manipulations (Table 2 second and third rows).

### Skin Conductance Responses

Skin conductance signals of each participant were filtered using a 1 Hz low pass filter and analyzed using a peak-to-base measure^32^,^33^,^34^,^35^,^36^,^37^,^38^. For each trial, we computed the difference between the maximum value detected in a 5 sec post-stimulus time window and the relative baseline (calculated as the average value of a 0.3 sec prestimulus time window)^32^,^33^,^34^,^35^,^38^.

The data passed the Kolmogorov–Smirnov test for normality and were analyzed using a one-way repeated-measures ANOVA and Duncan test for the within group effect of Visual Appearance (Full-Limb, Wire, m-Wrist, Plexiglass). Statistical analysis revealed that the SCR, evoked by a threatening stimulus over the virtual hand, was significantly modulated by the Visual Appearance (F(3, 54) = 3.992, p = 0.012; partial *η*^2^ = 0.182; power = 0.809; Fig. 2B). Within group comparisons revealed higher amplitudes of SCR when participants observed the threatening event over the Full-Limb (mean ± s.e.m.; 2.32 ± 0.47) relative to m-Wrist (1.34 ± 0.24; p = 0.013; d = 0.666) and Plexiglass condition (1.39 ± 0.25, p = 0.016; d = 0.565). Surprisingly, SCR’s amplitudes in the Wire condition (2.14 ± 0.45) did not differ from the Full-Limb condition (p = 0.632; d = 0.110) and were higher relative to the visual manipulations in which the virtual hand appeared disconnected to the rest of the body (m-Wrist, p = 0.036, d = 0.494; Plexiglass, p = 0.040, d = 0.445). No significant difference was found between m-Wrist and Plexiglass condition (p = 0.877, d = 0.041).

Finally, no correlation was found between subjective reports (mean of Hand-FO and Forearm-FO) and SCR measurements (Full-Limb: rho = −0.043, p = 0.86; Wire: rho = 0.025, p = 0.92; m-Wrist: rho = 0.047, p = 0.85; Plexiglass: rho = 0.017, p = 0.95).

## Discussion

In daily life we typically experience our own body in first-person perspective and we rarely doubt about the integrity, continuity of our body and sense of ownership over it. In the present study we used immersive virtual reality to investigate the influence of body visual discontinuity on the subjective feeling of ownership over a virtual limb and on the physiological response (Skin Conductance Responses, SCRs) elicited by a virtual stabbing. In line with previous studies^5^,^14^,^18^ we found that, in the absence of visuo-tactile or visuo-motor stimulation, the mere passive observation of an intact virtual limb from a 1PP elicited higher illusory FO relative to the observation of non-natural bodily appearances. Moreover, we showed that visual discontinuity between the virtual hand and forearm affected the physiological reactivity associated to a virtual threat. SCRs were stronger when the virtual hand was connected to the rest of the body (Full-Limb and Wire conditions) compared to other conditions (m-Wrist and Plexiglass). In other words, while the subjective measures were sensitive to the visual representation of the body the SCRs were affected by the continuity between the hand and the forearm.

Previous IVR studies highlighted the role of visuo-tactile and visuo-motor congruency^20^,^21^,^22^, visual perspective^15^,^16^,^17^,^18^ and visual continuity^23^,^24^,^25^ in eliciting an illusory ownership over the observed body. Importantly, two recent studies assessed the illusory FO combining visual continuity and visuo-tactile stimulation^26^,^31^. Perez-Marcos and colleagues^26^ showed that the FO over a virtual hand was highly dependent on synchronous visuo-tactile stimulation and the physical continuity between the virtual hand and shoulder^25^. Similarly, using an fMRI paradigm, Petkova *et al*.^31^ revealed an increased activation in multisensory areas when the stimulated body part appeared attached to the body^31^. We recently extended these findings by demonstrating that a small visual discontinuity between the virtual hand and forearm reduces the subjective illusory FO^18^ over the virtual hand also during a passive observation in 1PP when no visuo-tactile stimulation is applied.

The present study replicates our previous findings^18^ and confirms the importance of the visual integrity of the body in eliciting a subjective experience of ownership^3^,^4^,^39^,^40^. Moreover, our participants did not observe a projection of their real hand^28^ and did not move any finger^20^,^28^. Thus the observation of a possible visuo-motor congruence between the disconnected and connected body-parts has been prevented. This may explain the reduced FO over the whole limb (see^28^ for high ownership over a video-projected intact hand when participants are asked to tap with a connected finger) regardless of which specific part of the arm has been disconnected and extend previous knowledge about the relation between body ownership and body visual continuity.

There is a plethora of experimental observations highlighting that the illusory feeling of ownership over an external body is reflected by the activation of autonomic nervous system observed in the SCR^2^,^24^,^39^,^41^,^42^,^43^. Armel and Ramachandran^2^ first described that during a state of illusory FO elicited by a synchronous visuo-tactile stimulation on a rubber hand and the participant’s own hidden hand, an increase in SCRs occurred when a threatening stimulus was directed towards the rubber hand. Following the same line, many authors demonstrated an increase of SCR evoked by a threatening event over a full body^24^,^25^,^41^,^44^ or a rubber^43^,^45^, projected^42^,^46^, robotic^47^ and virtual hand^39^,^48^. Newport and Preston^28^ reported the only experimental evidence for threat-induced modulation of SCRs in conditions where a visual discontinuity was applied on the projected hand. In this study participants moved synchronously or asynchronously their video-projected finger that could appear normal or stretched to twice its normal length and detached from the hand. Independently from the visuo-motor congruency authors found reduced SCRs, evoked by a threatening event approaching the finger and the hand, when the finger appeared detached from the hand compared to the condition in which no discontinuity was applied^49^. Interestingly, we expand on this result by showing strong SCRs when the limb was connected to the rest of the body with both a natural (Full-Limb) and non-natural (Wire) visual appearance. Our data may suggest that visual continuity of the observed body is crucial for eliciting autonomic reactions to a threat even if the virtual limb is connected to the rest of the body by artificial means.

Note that in our study only the visual appearance of the avatar’s virtual limb was manipulated and no alteration of the peripersonal space, i.e. stretching the virtual limb^28^,^29^, or multisensory stimulation^26^ occurred. Importantly, we observed a discrepancy between the subjective ratings and SCR amplitudes during the Wire connection. In this case participants reported low levels of subjective FO; however, SCR amplitudes did not differ from the Full-Limb condition. Importantly, we adopted an experimental condition that emphasized the disconnection between the forearm and the hand (Plexiglass) in which we recorded low FO and low SCRs. This can exclude any possible bias of the attention-capturing or arousing effect induced by the observation of an object between two disconnected body parts. Moreover, our hand-forearm manipulation did not alter the visibility of the entire hand^42^,^50^,^51^ or the full-body^27^,^50^ but only the wrist joint appearance. Crucially, that SCR was comparable in the full limb and in the Wire condition (relative to the m-Wrist and Plexyglass conditions) rules out non-specific changes in the physiological reactivity. Rather, this result suggests that implicit reactivity may be linked to the possibility that any physical continuity allows the implementation of protective reactions to threat.

It is worth noting that the observation of rubber hands in anatomically incongruent posture alters the integration of multisensory signals^5^ and the elongation of the observed virtual limbs may extend the boundaries of the bodily space^29^. In contrast, our paradigm was not devised to explore the participant’s peripersonal space and cannot provide specific information on this issue. Thus, future studies using additional measures (i.e. proprioceptive drift, tactile sensitivity^52^) will be necessary to better understand the explicit-implicit relation between body and space representation.

Overall, the dissociation reported in the present study suggests that the visual appearance of the virtual limb, in terms of visual (dis)continuity, affects the feeling of ownership at the level of conscious appraisal, while a mere physical connection between the body parts may be enough to trigger autonomic reactions to threat. This suggests that multiple body representations and body reactivity to potential threats can be dissociated and that an interplay between distinct conscious and unconscious mechanisms may contribute to the detection of threatening approaching stimuli^49^.

Although it could be argued that the different modulation in FO and SCRs following our visual manipulations and the missing correlation between these measurements may reflect the weak relation between subjective and objective assessments of body ownership^45^,^53^,^54^, we cannot exclude that other factors may account for our results. There is evidence that bodily illusions^45^,^55^ and subjective FO and SCRs may be stronger for the left relative to the right hand^45^. Thus, the physiological recording from participants’ right hand and the absence of visuo-tactile stimulation lead participants to fully rely on the visual differences between the experimental conditions and might have reduced the possibility to find a correlation between the subjective and objective measures.

All in all, our study presents three major points of novelty. First, the absence of visual body information negatively affects the feeling of ownership even in conditions where, at variance with what happens in rubber-hand studies, no multisensory visuo-tactile and visuo-motor integration is required. Second, the physical continuity of a virtual limb seems to be fundamental for inducing a physiological reaction to a threatening event approaching the virtual hand. Third, our study shows a novel method to investigate implicit and explicit knowledge of body representation in healthy population using visual discontinuity paradigms in IVR in the absence of any spatial, posture or tactile mismatches between body and space. This approach may be relevant to better understand the neural and behavioural mechanisms responsible for the creation of a correct body representation in healthy subjects and in people with body-related disorders^32^,^56^. Our data suggest that multiple levels in the perception and knowledge of the body^57^ differently contribute to both implicit and explicit feeling of owning a body. We observed that the conscious inclusion of an artificial limb into one’s own body/self-representations requires a natural looking visual body appearance. In contrast, the implicit embodiment of body parts requires specific functional properties (e.g. physical continuity) that allow protective reactions to be effectively implemented (e.g. retracting the virtual hand before the stubbing). This phenomenon is reminiscent of dissociations between conscious and unconscious representations (e.g. the imperviousness of the motor system to visual illusions^58^ or the tendency to react, while walking in a wood, to a curved rod as though it were a snake^59^) where mainly the operational consequences matter. The reported dissociation showed that non biological wrist joint can reinstate an implicit body image and has important implications for understanding how building and maintaining a defensive self takes place.

## Methods

### Participants

Twenty healthy volunteers took part in the study (ten male; age mean ± SD, 28 ± 2.5). All participants were right-handed with normal visual acuity and were naıve as to the purposes of the experiment. The experimental protocol was approved by the ethics committee of the Fondazione Santa Lucia and was carried out in accordance with the ethical standards of the 1964 Declaration of Helsinki. All participants gave their written informed consent to take part in the study.

### Experimental Stimuli and setup

**Figure 1:**
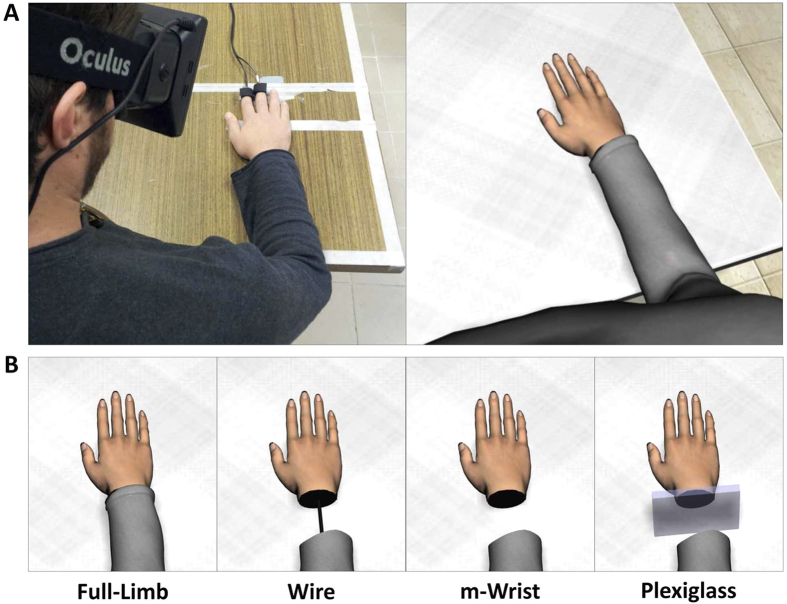
(**A**) Participants observed in 1PP through a HMD the virtual body in the same location and posture as the physical one; (**B**) The visual appearances of the avatar’s right limb: from left to right: a standard full arm (Full-Limb), a limb with a thin black rigid wire connecting the forearm and the hand (Wire), a limb with a missing wrist (m-Wrist) and a limb with a missing wrist with a Plexiglass panel placed in the blank space between the hand and the forearm (Plexiglass). Virtual models were created by the authors (see Experimental Stimuli and setup for details).

**Figure 2:**
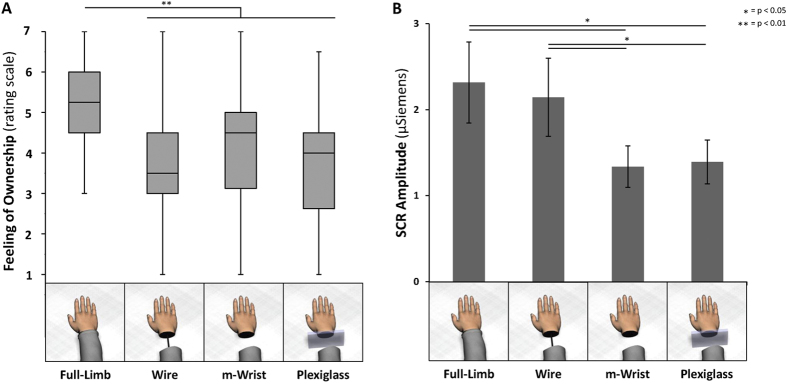
(**A**) Graphs represent the subjective ratings concerning the FO over the virtual hand and Forearm for each visual appearance. The horizontal black bars are the medians, and the boxes the interquartile ranges (IQRs). The whiskers stretch to the data points that are within the minimum and maximum score; (**B**) Graphs represent SCRs amplitude for each experimental condition.

The virtual scenario was designed using 3DS Max 2011 (Autodesk, Inc.) and implemented in XVR^60^ (http://www.vrmedia.it/en.html). It consisted in a large dining room and a virtual avatar sitting in front of a table placed in the center of the room (1:1 scale). The virtual avatar was created using Poser Pro 2010 library (Smith Micro, Inc.) and rendered in XVR. Participants passively observed the virtual body and the scenario from a first-person perspective (1PP) by means of an Head Mounted Display (HMD; www.oculusvr.com; Fig. 1A). The HMD has 110° field-of-view (diagonal FOV) with a resolution of 1280 × 800 (16:10 aspect ratio, 640 × 800 per eye) and an internal sensor for head tracking (3 degrees of freedom).

The avatar’s right limb had four different visual appearances (Fig. 1B): (i) a standard full limb (i.e. with hand connected to the rest of the body; Full-Limb); (ii) a limb with a thin black rigid wire connecting the forearm and the hand (Wire); (iii) a limb with a missing wrist (m-Wrist) and (iv) a limb with a Plexiglass panel placed in a blank space between the hand and the forearm (Plexiglass).

AD-Instruments PowerLab 8/35 and ML116 GSR Amplifier (providing a 75 Hz AC excitation with low constant-voltage of 22 mVrms) devices were used as signal amplifier with specific GSR sensors consisting of two bipolar finger electrodes. The sensors were applied on the distal phalanx of the index and middle fingers of the right hand. The signal was sampled at 1 KHz, recorded and analyzed using the software LabChart 7 (AD-Instruments, Inc.). In order to assess the degree to which participants experienced the illusory feeling of ownership over the virtual right hand and forearm, a 4-items questionnaire adapted from previous studies^1^,^18^ was used (see Table 1). Two items referred to the FO over the virtual hand (Q1 and Q2) while the remaining two items referred to the FO over the virtual arm (Q3 and Q4). The participants rated their experience on a 7-point rating scale (1 = no sensation; 7 = highest sensation). The order of the questions was counterbalanced across participants.

## Procedure

Participants were seated on a chair in front of a table and wore the HMD, through which they passively observed in 1PP the virtual body and the environment (Fig. 1A). The avatar’s size was adjusted to the individual dimensions of each participant.

The experiment started with an initial familiarization phase in which participants were invited to look around the environment and describe the virtual scenario (~100 sec)^23^. Then participants underwent 4 separate blocks, one for each hand-forearm visual manipulation, counterbalanced across subjects. At the beginning of each block participants were informed that a virtual knife could appear and hit the virtual right hand but nothing would happen to their real hand and were invited to relax and to remain as still as possible. No visible movements were noted during the entire block (including immediately after the virtual stabbing).

**Figure 3:**
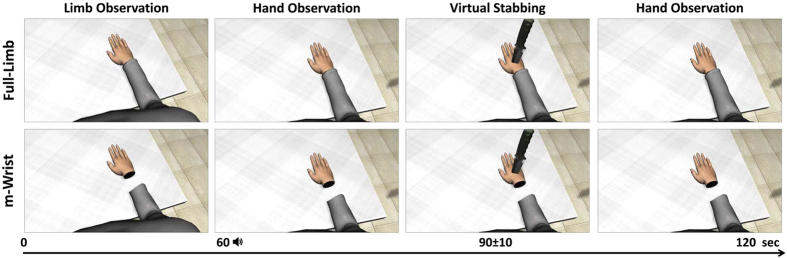
Sequence of events during Full-limb (upper) and m-Wrist condition (lower).

Each block lasted 120 seconds. Participants were informed that the block was divided in two continuous time windows signaled by a computer beep (Fig. 3). In the first half time window (from 0 to 60 sec) participants were asked to passively observe the virtual limb (arm, forearm and hand). Then, a computer beep sound signaled the beginning of the second half time window where participants were asked to focus their attention only on the virtual hand. A virtual stabbing randomly occurred within a 90 ± 10 sec time window after the starting of the block. The knife disappeared 5sec after the hit of the virtual hand.

At the end of each block a black screen covered the whole virtual scenario and participants were asked to verbally rate on a 7-point rating scale (1 = no sensation; 7 = highest sensation) the strength of the illusory ownership over the virtual hand and the virtual arm (see Table 3).

Finally, due to a technical limitation of the HMD, an indirect way to control whether participants were actually looking at the virtual hand/limb was used. In particular, for each block the experimenter continuously checked (i) the orientation of participants’ head (that had to be always directed toward their real hand) and (ii) the virtual scenario on the PC monitor (where the virtual hand/limb had to be always positioned in the center of monitor). If these conditions were not satisfied, participants were gently asked to look at the hand/limb body parts.

## Additional Information

**How to cite this article**: Tieri, G. *et al*. Body visual discontinuity affects feeling of ownership and skin conductance responses. *Sci. Rep*. **5**, 17139; doi: 10.1038/srep17139 (2015).

## Figures and Tables

**Table 1:** Model comparisons of subjective answers.

Model	*AIC*	*BIC*	*Log Likelihood*	*p*
Constant	705.34	744.65	−339.67	
Visual Appearance	653.72	702.10	−310.86	<0.001
Visual Appearance + Body Part	655.72	707.12	−310.86	0.95
Visual Appearance * Body Part	659.70	720.18	−309.85	0.57

AIC = Akaike information criterion, BIC = Bayesian information criterion.

**Table 2:** Planned contrasts (Helmert coding) of the winning model with Visual Appearance as single predictor.

Contrasts	*Estimate*	*odds*	*z*	*p*
(no-W-Limb, Plexy, Rigid-Wire) vs Full Limb	0.68	0.51	7.01	<0.001
(Plexy, Rigid-Wire) vs no-W-Limb	−0.10	1.11	0.86	0.39
Rigid Wire vs Plexy	0.35	0.71	1.71	0.09

**Table 3:** Questions assessing the perceived feeling of body ownership over the virtual hand (Hand-FO) and Arm (Arm-FO) using a 7-point rating scale (1 = no sensation; 7 = highest sensation).

Index	Item	
Hand-FO	*Q1*	I felt as if I were looking at my own hand
	*Q2*	I felt as if the virtual hand were part of my body
Arm-FO	*Q3*	I felt as if I were looking at my own arm
	*Q4*	I felt as if the virtual arm were part of my body
